# Social network analysis of international scientific collaboration on psychiatry research

**DOI:** 10.1186/1752-4458-9-2

**Published:** 2015-01-03

**Authors:** Ying Wu, Zhiguang Duan

**Affiliations:** School of Public Health, Shanxi Medical University, South Xinjian Road, Taiyuan, Shanxi China

**Keywords:** Psychiatry, Collaboration, SNA

## Abstract

**Background:**

Mental disorder is harmful to human health, effects social life seriously and still brings a heavy burden for countries all over the world. Scientific collaboration has become the indispensable choice for progress in the field of biomedicine. However, there have been few scientific publications on scientific collaboration in psychiatry research so far. The aim of this study was to measure the activities of scientific collaboration in psychiatry research at the level of authors, institutions and countries.

**Methods:**

We retrieved 36557 papers about psychiatry from Science Ciation Index Expanded (SCI-Expanded) in web of science. Additionally, some methods such as social network analysis (SNA), K-plex analysis and Core-Periphery were used in this study.

**Results:**

Collaboration has been increasing at the level of authors, institutions and countries in psychiatry in the last ten years. We selected the top 100 prolific authors, institutions and 30 countries to construct collaborative map respectively. Freedman, R and Seidman, LJ were the central authors, Harvard university was the central institution and the USA was the central country of the whole network. Notably, the rate of economic development of countries affected collaborative behavior.

**Conclusion:**

The results show that we should encourage multiple collaboration types in psychiatry research as they not only help researchers to master the current research hotspots but also provide scientific basis for clinical research on psychiatry and suggest policies to promote the development of this area.

## Background

Mental disorder is harmful to human health and effects social life seriously [1]. According to a recent survey of the World Health Organization, it has been estimated that mental disorder ranks the first in terms of dis-ability life years (DALYs) which will surpass that of cardiovascular disease, respiratory system disease and malignant tumor [2–4]. This troubling situation has brought a rigorous challenge for the psychiatry researchers to prevent and control mental disorder. With the interdigitating of subjects in biomedicine field, no single one can finish all the specialist tasks. Thus, research collaboration becomes the indispensable choice for progression in the biomedicine field because it will improve communication, the sharing of competence and production of new scientific knowledge. The most evident form of collaboration is co-authorship, which is a frequent and reliable target of research collaboration [5]. So, studying the co-authored phenomenon of the academic papers can help us understand the types, characteristics and law of scientific research collaboration better to make research plan and organize the implementation of the scientific research collaboration in order to improve the quality and efficiency of scientific research. Scientific collaborative network is a relationship network in which the researchers of one field collaborated each other to research and write papers [6]. In recent year, many scholars have devoted themselves to collaboration in different scientific fields. In 2001, the American scholar Newman began to study the structure of scientific collaboration networks of fields such as biomedicine, physics and computer science [7]. Liu XM, Bollen J et al. analyzed the collaboration pattern of digital library by co-authorship network in 2005 [8]. Hou HY, Kretshmer H studied on co-authorship network of scientometric by using the data from SCI in 2008 [9]. In 2011 and 2012, Yu Q, Duan ZG et al. used the method of co-authorship to analyze collaboration in Chinese oncology and cardiology & cardiovasology field [10, 11].

However, there have been few research on scientific collaboration in psychiatry research so far. Therefore, we designed this study to measure scientific collaboration activities at the level of authors, institutions and countries respectively in psychiatry research.

## Methods

We selected 36557 documents on ten psychiatric journals with top Impact Factor (IF) from Science Citation Index Expanded (SCI-Expanded) in web of science during 1983 to 2012. These ten psychiatric journals were from JCR (Journal Citation Reports) in web of science in 2012 (Table 1). The date of each bibliographic record contained title, author names, abstract, key words and references, ect. The date included 83469 authors, 5182 institutions and 107 countries. A paper co-authored by authors from more than one institution was considered inter-institutional collaboration and a paper co-authored by authors from different counties was classified as inter-national collaboration.

**Table 1:** **10 representative journal in psychiatry field**

Rank	Journal title	Impact factor
1	Molecular Psychatry	14.897
2	American Journal of Psychiatry	14.721
3	Archives of General Psychiatry	13.772
4	Biological Psychiatry	9.247
5	World Psychiatry	8.974
6	Neuropsychopharmacology	8.678
7	Schizophrenia Bull	8.486
8	Psychotherapy and Psychosomatics	7.23
9	Journal of the American academy of Child and Adolescent Psychiatry	6.97
10	British Journal of Psychiatry	6.606

Social network analysis (SNA) is a kind of structure analysis method developing in many research fields which focuses on the relationship research and is mainly used to describe and measure the relationship and information individually [12, 13]. Theories of SNA have been proved to be successful in studies of scientific collaboration network [14, 15], In this study, we used SNA to analyze the collaborative connection among authors, institutions and countries in psychiatry research. Centrality, which reflects status and rights of activities in their social network, is one of the most important content in network analysis. There are three common centrality measures: degree centrality, betweenness centrality and closeness centrality. In the collaborative network, degree centrality is equal to the number of nodes that connect with a central node. That is, if an author/institution/country has the highest degree centrality, it is considered a central author/institution/country in the collaboration network. Betweenness centrality is the number of the shortest paths that pass through a given node [9]. In our study, the highest betweenness centrality would indicate that an author/institution/country possesses and controls a great deal of research resource. Finally, Closeness centrality of a node is equal to reciprocal of the total distance from this node to all other nodes. It means the closer a node is to all other nodes, the higher is its closeness centrality. The lowest closeness centrality indicates an author/institution/country is at the core position of the entire network. UCINET and Netdraw were used to identify and visualize authors’, institutions’ and countries’ collaborative network structures [16, 17].

There were 36557 papers about psychiatry retrieved from these ten journals during 2003–2012. Among them, the total number of co-authorship papers was 29430. From the Table 2, the total number of papers has increased from 2754 in 2003 to 3029 in 2012 and the total number of co-authored papers has increased from 2217 in 2003 to 2297 in 2012. It suggested that the scale of collaboration was related with the output of scientific research positively.

**Table 2:** **Co-authored papers on psychiatry research**

Year	Total papers	Co-authored papers
2003	2754	2217
2004	3849	3085
2005	4919	4072
2006	3949	3229
2007	4387	3698
2008	3171	2491
2009	3984	3247
2010	2872	2182
2011	3643	2912
2012	3029	2297

## Results

### Analysis on authors ’collaboration

Achievements in scientific research are published in the form of papers and the status of co-authorship in papers reflects collaboration among authors. M.smith was one of the scientists who studied the growth of co-authorship papers made by multi-author and viewed co-authorship of papers as a importance scientometrics indicator of researching on collaboration among authors [18].

**Figure 1:**
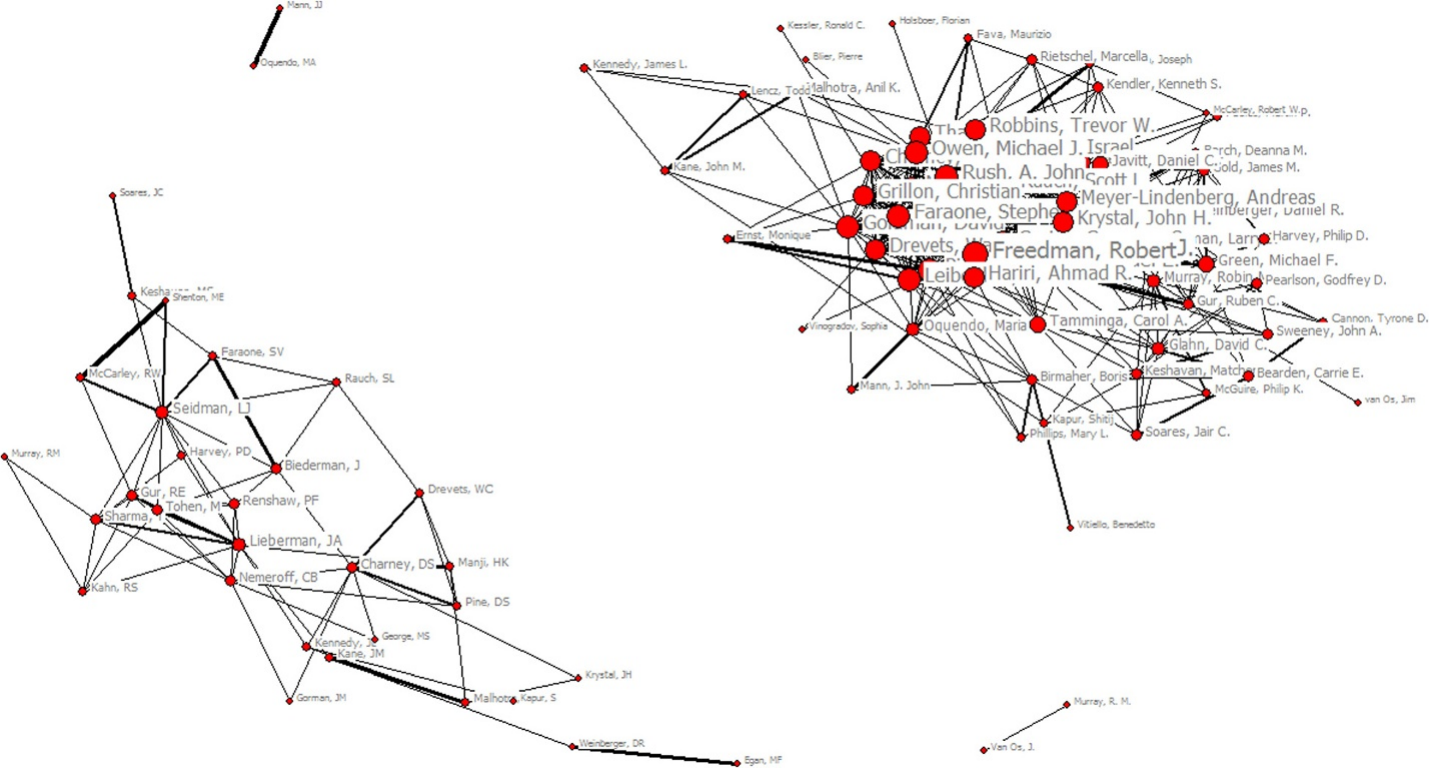
**The structure map of collaboration network among authors on psychiatry research.**

In order to show the main co-authorship structure of the network, we selected the top 100 prolific authors during 2003 to 2012 in this study. This threshold resulted in the top 100 prolific authors who must publish 43 co-authorship papers. (Three authors who have not cooperated with other authors were deleted), so the Figure 1 was a co-authorship map made up of the top 100 authors visualizing the structure of authors’ collaboration network. The map was composed of four sub-networks which are not connected with each other. The line value and the distance between two vertices represent the collaborative strength, while thickness of the line represents the number of co-auhorship papers. In this authors’ collaboration network, the highest degree centrality of Pine, DS was 145 indicated he had 145 collaborators, so he was the most key author of the co-authorship network. Seidman, LJ had got the highest betweeness centrality which indicated that he possessed and controlled a great deal of research resource. Fava, M had the lowest closeness centrality which indicated he was in a core position of the whole network (see Table 2). In collaborative network, betweeness centrality reflects the author’s function. The lack of author with the highest betweeness centrality lead to connection interruption of collaborative network. That Seidman, LJ had got the highest betweeness centrality indicated he had the power to control collaborative relationship. In collaborative network, the closer one author is to the other author, more easily are information communication and research collaboration. Freedman, R had the lowest closeness centrality which indicated that he possessed and controlled a great deal of research and was in a core position of the whole network (see Table 3).

**Figure 2:**
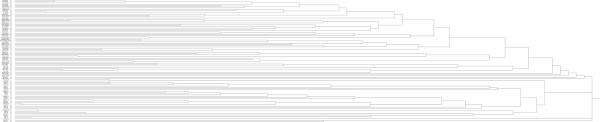
**Dendrogram of the prolific authors.**

Hierarchical clustering usually categorizes prolific authors and creates a hierarchy of clusters which can be represented in a tree structure called a dendrogram. Through Hierarchical clustering analysis, we got Figure 2 and we divided 97 authors into 3 sub-networks (see Figure 1). The largest sub-network included 60 nodes and 754 lines. In this subnetwork, the average path length was 2.091 and the average clustering coefficient was 0.651 which indicated obvious clustering effect and characteristics of small world. In this subnetwork, Freedman, R had the highest centrality degree and his research direction was mainly child and adolescent psychiatry. Siever, LJ had the second centrality degree and his research direction was mainly pathophysiology of child and adolescent schizophrenia. Carter, CS had the third centrality degree and his research direction was mainly cognitive dysfunction of schizophrenia. The second sub-network included 35 nodes and 166 lines. In this sub-network, the average path length was 2.545 and the average clustering coefficient was 0.502 which indicated obvious clustering effect and characteristics of small world. In this subnetwork, Seidman, LJ had the highest centrality degree and his research direction was mainly brain imaging of schizophrenia. Liberman, JA had the second centrality degree and his research direction was mainly on drug treatment of schizophrenia. Sharma,T had the third centrality degree and his research direction also mainly on drug treatment of schizophrenia. The third sub-network only included Egan, MF and Kendler, KS and their research direction was molecular genetics of psychiatry.

**Table 3:** **Top 10 authors on centrality measures in collaborative network**

Degree	Score	Betweenness	Score	Closeness	Score
Pine, DS	145	Seidman, LJ	201.652	Freedman, R	4091
Weinberger, DR	124	Faraone, SV	171.760	Faraone, SV	4093
Freedman, R	92	Rush, AJ	150.512	Siever, LJ	4093
Sharma,T	86	Murray, RM	138.624	Carter, CS	4094
Leibenluft, E	85	Keshavan, MS	134.467	Pine, DS	4095
Seidman, LJ	84	Hariri, AR	133.269	Hariri, AR	4095
Mattay, VS	84	Lieberman, JA	126.846	Leibenluft, E	4096
Faraone, SV	78	Freedman, R	108.138	Rush, AJ	4097
Callicott, JH	75	Pine, DS	93.802	Meyer-Lindenberg, A	4098
Gur, RE	72	Carter, CS	83.672	Gelernter, J	4099

A K-plex is a maximal sub-network in which each node has at least connected with other nodes except these K nodes directly within the sub-network. It is widely used in collaborative network which is undirected and has more value network. In the first, determine the condensation degree of subgroup. The critical value ‘C’ is bigger, the condensation degree of subgroup is stronger. If the value between ‘g’ nodes to ‘g-k’ nodes all at least not less than ‘c’ in a subgroup, we called this subgroup ‘c’level K-plex [19]. In order to exclude the phenomenon of the fewer number of collaboration, ‘C’ was determined to be ‘4’. It indicated the authors who collaborated with others less than 4 time would no longer appear in K-plex and these subgroups were higher cohesive in which the members were keeping a relatively close relationship. By using UCINET there were totally 776 ‘K-2’ K-plexs which collaborative frequency was greater than 4. It indicated the number of collaboration between any author in ‘K-2’ K-plex and other two authors was no less than 4 time. Pine, DS appeared in 449 ‘K-2’ K-plexs and Weinberger, DR appeared in 319 ‘K-2’ K-plexs.

### Analysis on institutions’ collaboration

**Figure 3:**
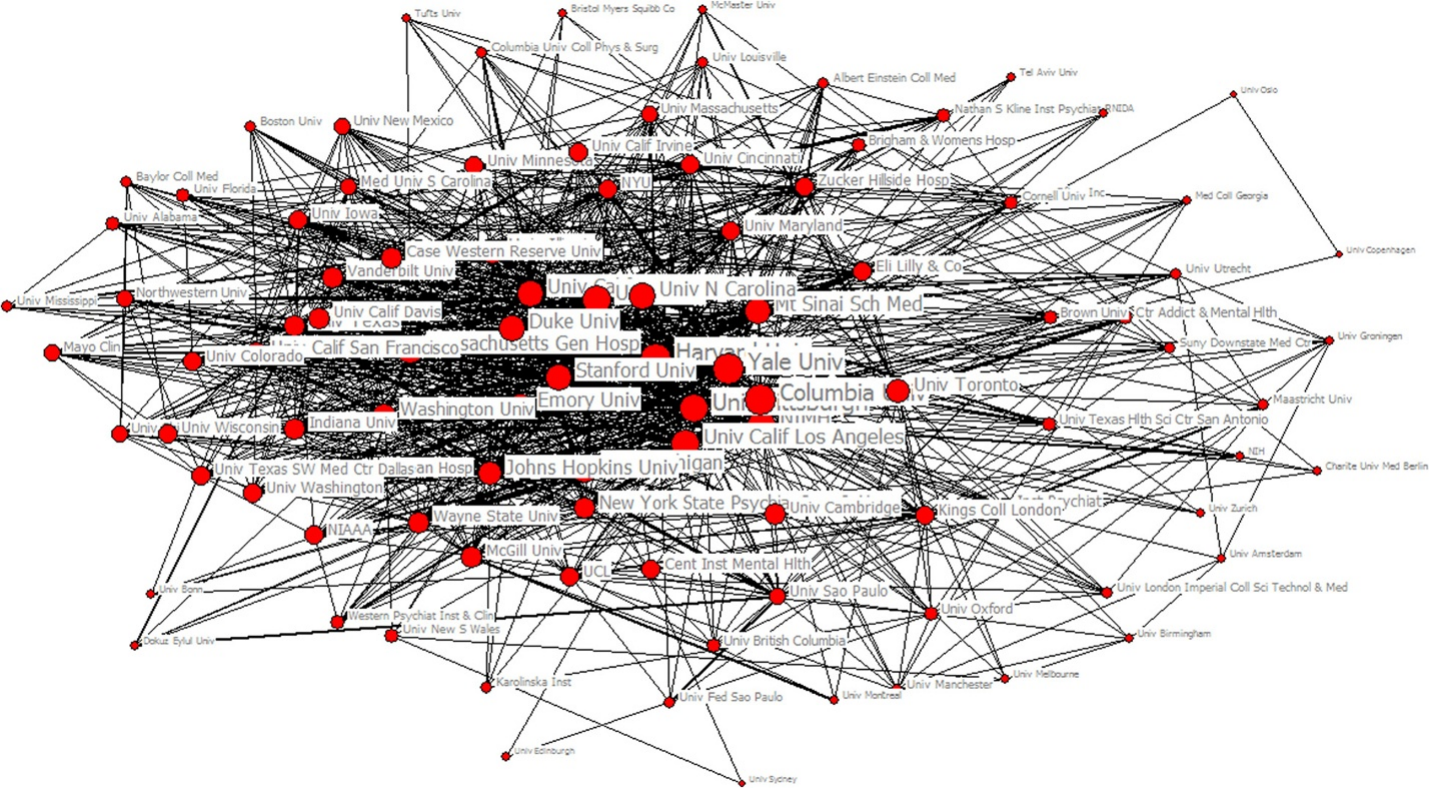
**The structure map of the institutional collaboration network on psychiatry research.**

**Figure 4:**
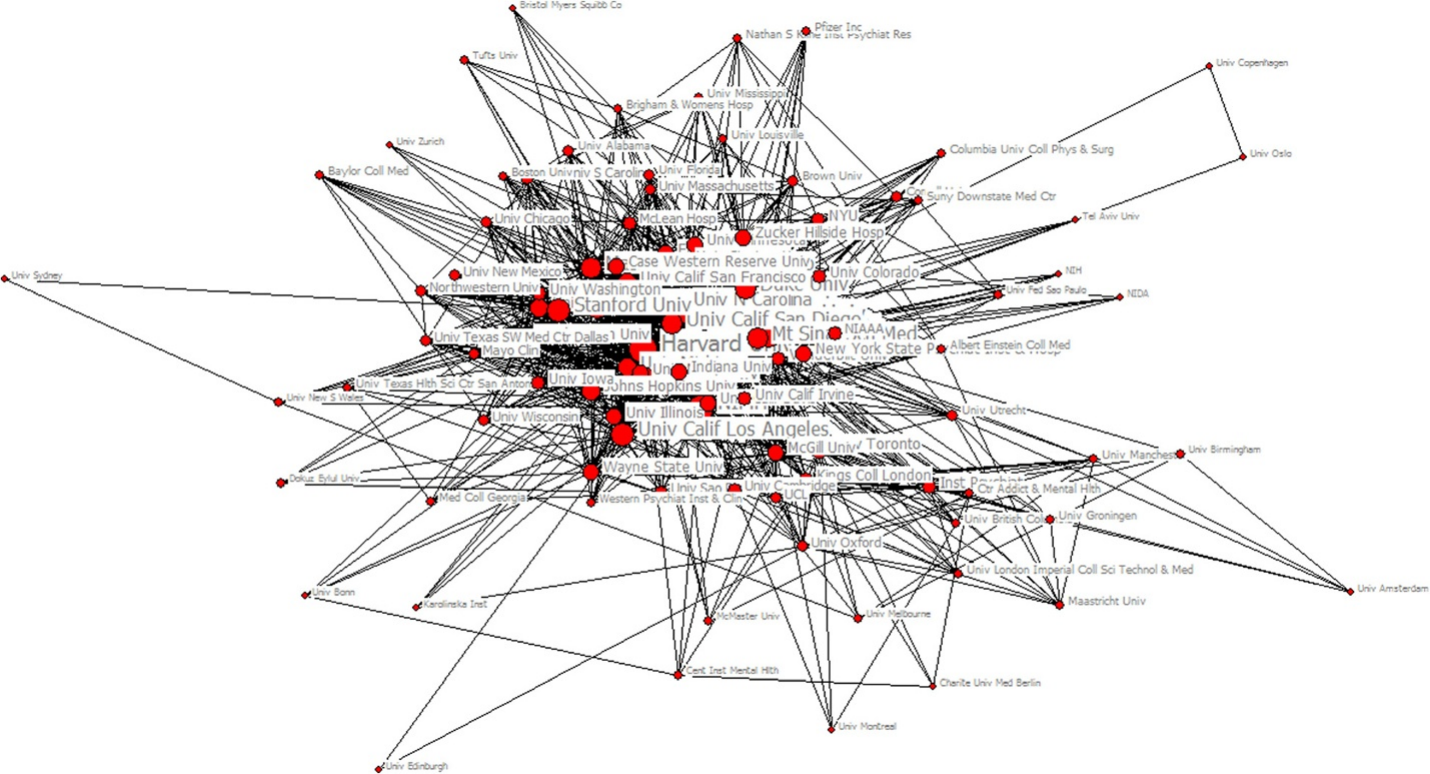
**The core**-**periphery structure map of the institutional collaboration network on psychiatry research.**

Since the 1920s,with the rapid development on the scale and scope of research collaboration, the collaborative papers among institutions increased 46% [20]. Analysis on relationship network of academic institutions in research collaboration is of great significant to research mechanism, influence factors and academic information exchange model in scientific collaboration. There were 19475 papers which belonged to inter-institution collaboration among 36557 papers from SCI during 2003 to 2012. The number of papers has increased from 1072 in 2003 to 1598 in 2012. These papers covered 5182 actual institutions and the appearing frequency of institutions was totally 36653.The largest collaboration in our sample involved 47 institutions. Seen from Table 4 which described the annual change in institutions, the appearing frequency of institutions grew significantly in 2005 and 2009 and the number of actual institutions increased in the two years while the achievements in scientific research rose respectively in 2005 and 2009. It suggested that the scale of collaboration was related with the output of scientific research positively. We selected the top 100 institutions with appearing frequencies more than 57 to form a map visualizing the structure of institutions’ collaboration network in the field of psychiatry during 2003 to 2012 (see Figure 3). The size of node represents centrality in collaborative network. Harvard University had the highest degree centrality, and Yale University had the highest betweenness centrality and the lowest closeness centrality (see Table 5). It shows Harvard and Yale University were in high level of collaboration. The distance and thickness of the line between two nodes represent their collaborative strength and the number of collaborative papers respectively. From Figure 3, National Institutes of Health (NIH), Havard University and Yale University were in the center of collaborative network which had a fundamental impact on the development of psychiatry; while University of Zurich and University of Louisville were on the edge of collaborative network. Relative to the institutions in the center, scientific research strength of institutions on the edge were slightly inferior and collaboration closely among the institutions in the center reflected obvious ‘center effect’ in the process of co-authorship while the institutions on the edge collaborated looser. Analysis on Core-Periphery is the quantitative study of various network. We applied this method to collaborative network and used UCINET 6.0 to calculate ‘correlative value of collaborative network’ being 0.628. From Figure 4, we detected the polarized Core-Periphery structure was very obvious. In other words, collaborative network showed obvious regional characteristics and firstly select research institution in the close geographical position. That the highest collaborative frequency of Harvard University and Yale University indicated these two research institutions have a close research collaborative relationship.

**Table 4:** **Annual institutional change on psychiatry research**

Year	Frenquency of institutions	Actual institutions	Number of papers
2003	2099	707	2778
2004	3302	900	3864
2005	4037	1052	4944
2006	3565	951	3884
2007	4114	1103	4907
2008	3175	916	3191
2009	5292	1411	3959
2010	2592	833	2889
2011	5046	1409	3565
2012	3431	1066	3068

**Table 5:** **Top 10 institutions on centrality measures in collaborative network**

Degree	Score	Betweenness	Score	Closeness	Score
Harvard University	724	Yale University	258.013	Yale Univerity	116
Columbia University	571	Harvard University	185.469	Harvard University	117
University of Pittsburgh	533	University of Pittsburgh	181.224	Columbia University	119
Yale University	513	Columbia University	168.600	University of Pittsburgh	120
The National Institute of Mental Health (NIMH)	473	The National Institute of Mental Health (NIMH)	145.842	The National Institute of Mental Health (NIMH)	123
Penn University	379	Univerity of California- Los Angeles	130.578	Univerity of California- Los Angeles	125
Massachusetts General Hospital	366	Toronto University	113.269	Penn University	126
Univerity of California- Los Angeles	365	Penn University	105.638	Duke University	129
University of North Carolina	350	Stanford University	99.105	Stanford University	129
University of California-San Diego	336	University of California-San Diego	89.141	Mount Sinai School of Medicine	131

### Analysis on countries’ collaboration

**Figure 5:**
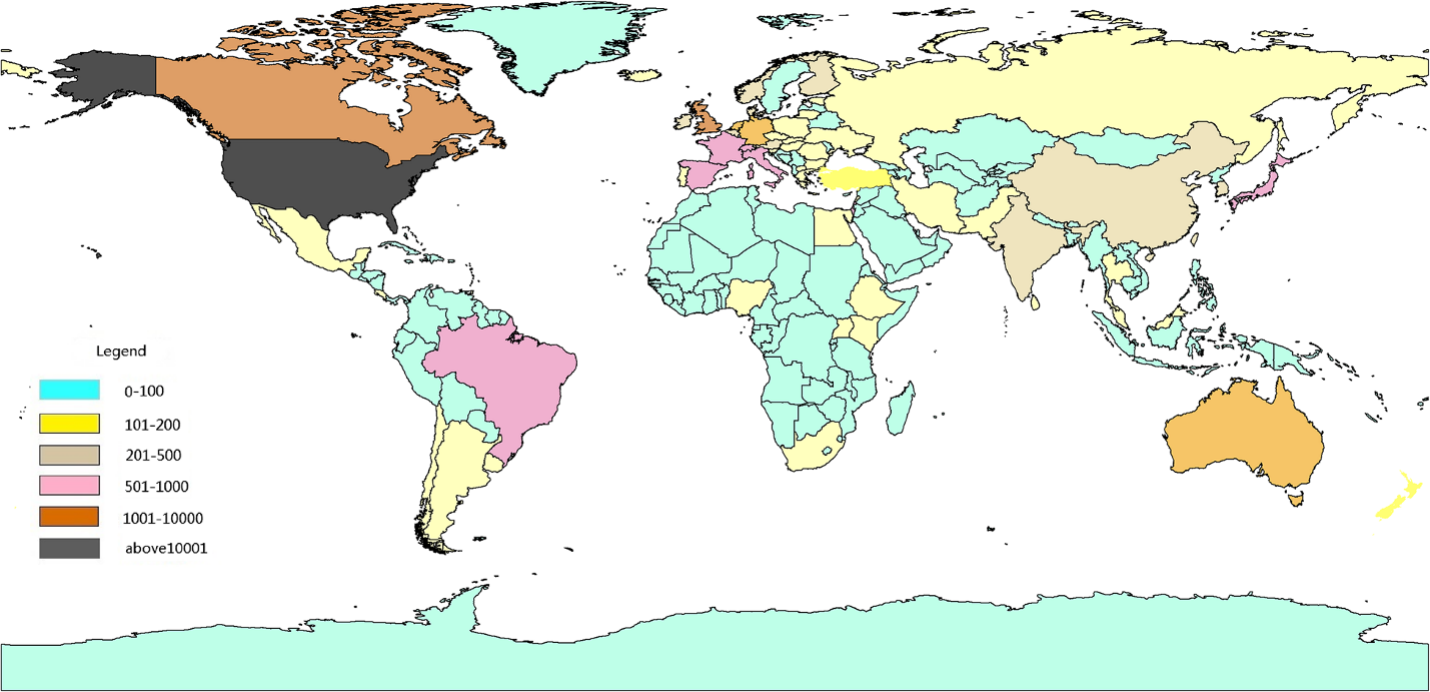
**Country distribution of global psychiatric papers.**

**Figure 6:**
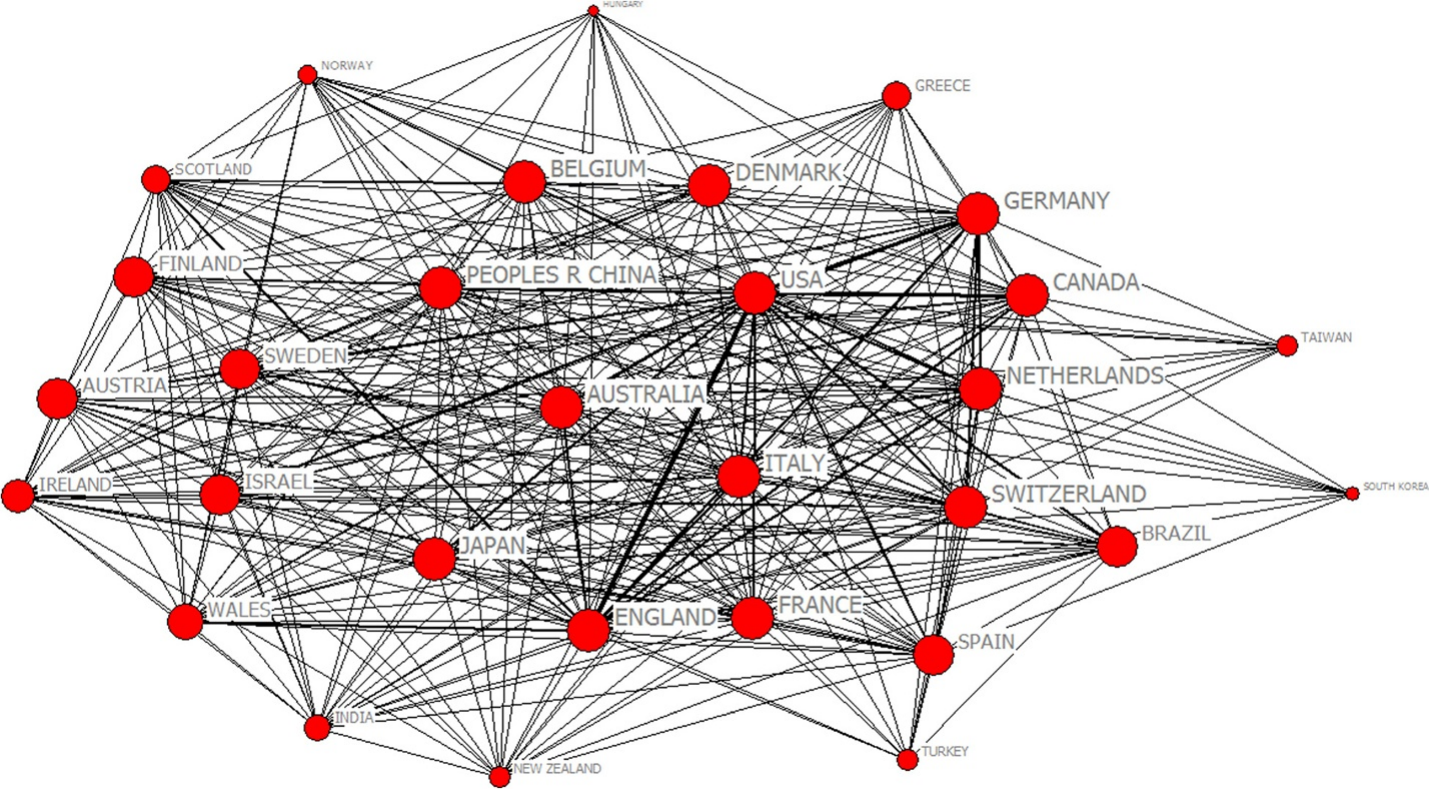
**The structure map of collaboration network among countries on psychiatry research.**

**Figure 7:**
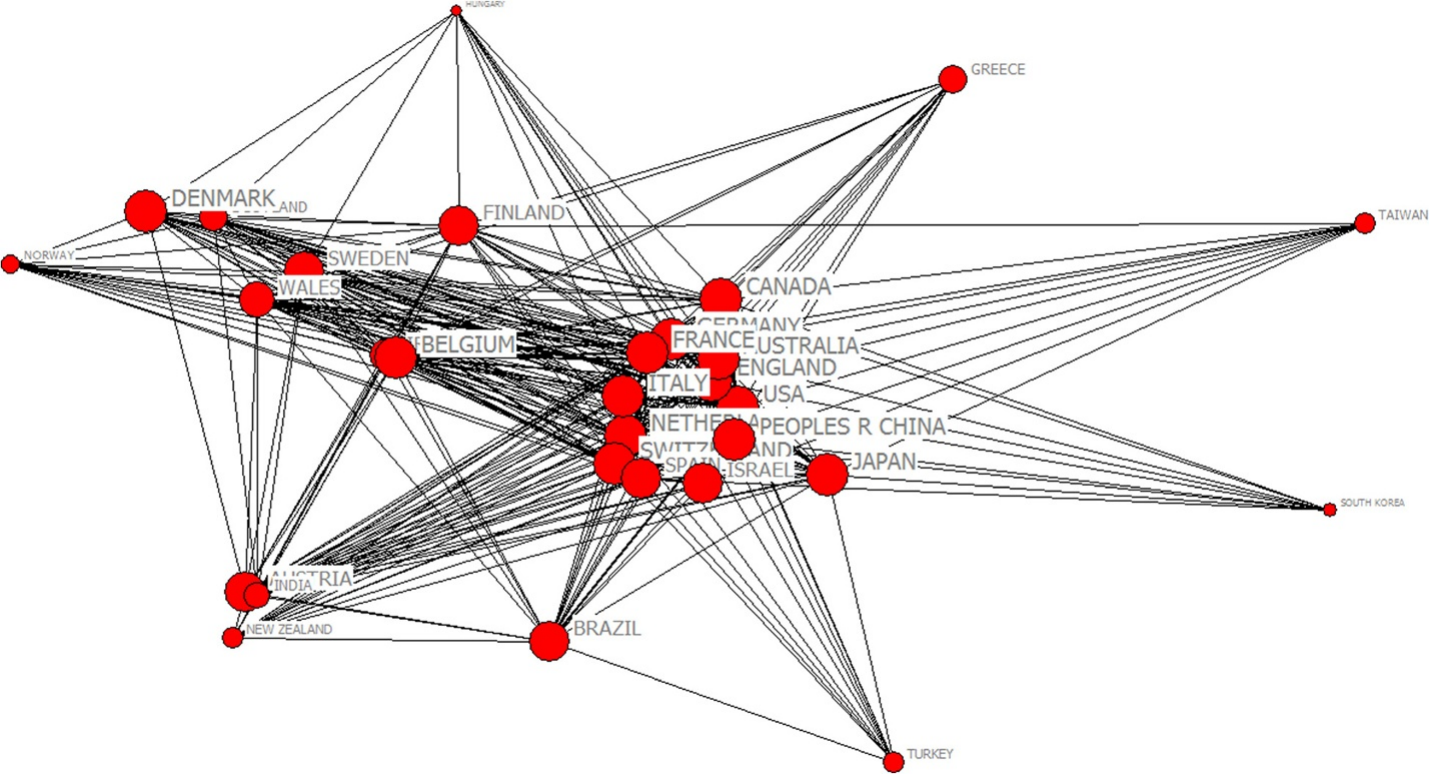
**The core-** **periphery structure map of collaboration network among countries on psychiatry research.**

Studies showed that research papers produced by international collaboration had larger impact [21]. In the 1990s, collaborative papers among countries increased by 115% within the amount of research paper from SCI [20]. During 2003 to 2012, the total number of countries was 107. From Figure 5, the countries with highest productivity include USA, England and Canada. There were 12808 papers in psychiatry research originated from USA than other countries and 60.6% of the total number of documents. There were 99 papers in China, ranked the 19th place. We chose the top 30 countries with appearing frequencies more than 86. Figure 6 was the map of the scientific collaboration of the most productive countries in the world. The network included 30 nodes and 1032 lines. In this network, the average path length was 1.076 and the average clustering coefficient was 37.572 which indicated obvious clustering effect and characteristics of small world. We applied Core-Periphery analysis and used UCINET 6.0 to calculate correlative value of collaborative network being 0.899. From Figure 7, we detected the polarized Core-Periphery structure was very obvious.

We analyzed international scientific collaborative effect on national scientific through the correlation between the number of corresponding nodes and scientific research achievements. The national names, the number of ties and the product of papers which the 30 round nodes corresponded with were listed in the Table 6. We found that there were 21106 papers produced by 30 countries which collaborated more frequently accounting for 58% of the total output in 107 countries. From the Table 6, that the international order of these 30 countries was identical with their research output showed that international scientific collaboration had great influence on output of scientific research in this field. Scientific collaboration was basically correlated with the output of papers positively and the countries which had frequent scientific collaboration had larger research output.

**Table 6:** **The relation between international collaboration and scientific papers**

Collaboration		Country	Production	
Ranks	Ties		Papers	Ranks
1	30	USA	12808	1
2	30	England	2006	2
3	30	Canada	724	4
4	30	Netherlands	461	6
5	30	Germany	1146	3
6	30	Italy	230	10
7	30	France	291	9
8	30	Australia	464	5
9	30	Switzerland	195	14
10	30	Japan	196	12
11	30	Belgium	90	22
12	30	China	99	21
13	29	Sweden	190	15
14	29	Brazil	300	8
15	29	Israel	320	7
16	29	Spain	200	11
17	29	Denmark	144	19
18	29	Finland	73	25
19	29	Austria	78	24
20	28	Wales	151	16
21	27	Ireland	148	17
22	26	Scotland	196	13
23	26	Greece	25	30
24	25	India	148	18
25	24	New Zealand	45	28
26	24	Taiwan	79	23
27	23	Turkey	69	26
28	22	Norway	66	27
29	21	Korea	134	20
30	20	Hungry	30	29

From centrality analysis (see the Table 7). That the highest degree centrality of USA was 5783 and the highest betweenness centrality of USA was 1.621, while the lowest closeness degree of USA was 29 showed USA was the center of international scientific collaboration network in psychiatry field in the world. The large number of research output maked USA a major producer of international publications.

**Table 7:** **Top 10 countries on centrality measures in collaborative network**

Degree	Score	Betweenness	Score	Closeness	Score
USA	5783	USA	1.621	USA	29
England	3357	England	1.621	Germany	29
Germany	2097	Germany	1.621	England	29
Netherlands	1505	Italy	1.621	Australia	29
Canada	1487	Australia	1.621	Canada	29
Italy	1365	Canada	1.621	Italy	29
France	1063	France	1.621	France	29
Australia	994	Spain	1.621	Spain	29
Switzerland	932	Switzerland	1.621	Switzerland	29
Spain	930	Japan	1.621	Japan	29

## Discussion

Nowdays, with the development of economy and the increasing of social competitive pressure, the number of mental patients is growing up dramatically. Because of the diversity and complexity of this disease, scientific collaboration plays an indispensable role for progress of depression. Collaboration has increased at the levels of authors, institutions and countries supported by many studies [22–24]. Unfortunately, few publications about scientific collaboration in psychiatry research were reported. This study chose bibliographic date about psychiatry retrieved from the web of science during 2003 to 2012 to construct and analyze the scientific collaboration structure of psychiatry in the world at the level of authors, institutions and countries based on SNA and found that the scientific collaboration was the first factor to boost the rapid development of this area.

From view of publications in psychiatry field during 2003 to 2012, more than 80% of the papers was published by two or more collaborative authors and the output of achievements in scientific research by way of collaboration was consistent with the total output. This indicated that collaboration among authors to complete research publications has been the main research method. From the results of centrality analysis, Freedman, R and Seidman, LJ were the central authors of the whole network which indicated that they were the most influential persons in the field of psychiatry research in the world. According to it, we can easily select the leader of this field of learning. In the era of knowledge economy, as the most important economic factors, the intellectual resources become more and more obvious. So, international scientific collaboration laid a foundation for selecting the subject leader.

From the level of multi-institutional collaboration, the number of papers which collaborated among institutions have accounted for more than half of the total papers. The universities, research institutions and hospitals were the main current research institutions in this field, especially the universities were the absolute main force. With actual collaborative institutions increasing, the output of achievements in scientific research was on the rise according to the date from 2003 to 2012 which showed the output of scientific publications kept pace with actual collaborative institutions. That some research institutions which were devoted to research on psychiatry repeated greatly showed their research ability was gradually strengthening. Harvard and Yale Universities’ centralities were the highest which indicated they possessed and controlled a great deal of research resource, so they became the central of multi-institutional collaboration in psychiatry field all over the world. From the analysis on Core-Periphery structure, academic institutions in the process of co-authorship reflected obvious ‘center effect’ and because there were much collaboration among the famous university, it appeared the phenomenon of ‘elite universities assembling’. In other words, collaboration which was mainly in the form of institutions within the same country and the other research institutions need to collaborate with institutions which collaborated closely to strive for the more scientific research resource showed geographical characteristics.

From the level of multi-national collaboration, USA which centrality was the highest was in the most central position. Judging from analysis above, each of country’s scientific collaboration was basically correlated with its output of scientific research positively, but there was still a gap in psychiatry field and international scientific collaboration was relatively limited in China. From the analysis on Core-Periphery structure, developed countries such as USA and England collaborated closely. It showed that the ability of international collaboration and the output of scientific research were the highest all over the world was closely related with the rate of economic development which affect the collaboration behavior. Higher income countries prefer to collaborate with each other and lower income countries prefer to collaborate with higher incomes in order to yield high quality productions.

## Conclusion

This study described the collaborative behaviors in psychiatry research at the level of authors, institutions and countries. Collaboration research can help to select the leader of this subject. Collaboration can offer scientific evidences and reasonable suggestions as the basis of making polices to guide finance psychiatry research in the future.
